# Quadruplex-forming oligonucleotide targeted to the *VEGF* promoter inhibits growth of non-small cell lung cancer cells

**DOI:** 10.1371/journal.pone.0211046

**Published:** 2019-01-25

**Authors:** David Muench, Francine Rezzoug, Shelia D. Thomas, Jingjing Xiao, Ashraful Islam, Donald M. Miller, Kara C. Sedoris

**Affiliations:** 1 Department of Immunobiology, University of Cincinnati, Cincinnati, Ohio, United States of America; 2 James Graham Brown Cancer Center, Department of Medicine, University of Louisville, Louisville, Kentucky, United States of America; 3 Faculty of Medicine, University of Tabuk, Tabuk, Saudi Arabia; 4 Department of Physiology, University of Louisville, Louisville, Kentucky, United States of America; Universita degli Studi di Parma, ITALY

## Abstract

**Background:**

Vascular endothelial growth factor (VEGF) is commonly overexpressed in a variety of tumor types including lung cancer. As a key regulator of angiogenesis, it promotes tumor survival, growth, and metastasis through the activation of the downstream protein kinase B (AKT) and extracellular signal-regulated kinase (ERK 1/2) activation. The *VEGF* promoter contains a 36 bp guanine-rich sequence (VEGFq) which is capable of forming quadruplex (four-stranded) DNA. This sequence has been implicated in the down-regulation of both basal and inducible *VEGF* expression and represents an ideal target for inhibition of *VEGF* expression.

**Results:**

Our experiments demonstrate sequence-specific interaction between a G-rich quadruplex-forming oligonucleotide encoding a portion of the VEGFq sequence and its double stranded target sequence, suggesting that this G-rich oligonucleotide binds specifically to its complementary C-rich sequence in the genomic *VEGF* promoter by strand invasion. We show that treatment of A549 non-small lung cancer cells (NSCLC) with this oligonucleotide results in decreased VEGF expression and growth inhibition. The VEGFq oligonucleotide inhibits proliferation and invasion by decreasing *VEGF* mRNA/protein expression and subsequent ERK 1/2 and AKT activation. Furthermore, the VEGFq oligonucleotide is abundantly taken into cells, localized in the cytoplasm/nucleus, inherently stable in serum and intracellularly, and has no effect on non-transformed cells. Suppression of *VEGF* expression induces cytoplasmic accumulation of autophagic vacuoles and increased expression of LC3B, suggesting that VEGFq may induce autophagic cell death.

**Conclusion:**

Our data strongly suggest that the G-rich VEGFq oligonucleotide binds specifically to the C-rich strand of the genomic *VEGF* promoter, via strand invasion, stabilizing the quadruplex structure formed by the genomic G-rich sequence, resulting in transcriptional inhibition. Strand invading oligonucleotides represent a new approach to specifically inhibit *VEGF* expression that avoids many of the problems which have plagued the therapeutic use of oligonucleotides. This is a novel approach to specific inhibition of gene expression.

## Background

Vascular Endothelial Growth Factor (VEGF) plays a key role in tumor cell growth; causing increased proliferation, angiogenesis, and metastasis in a variety of tumor types including lung cancer.[1, 2] Expression of *VEGF* is primarily regulated at the transcriptional level and its expression can be induced physiologically by tumor hypoxia, hypoglycemia, loss of tumor suppressor genes, or by activation of growth factor signaling cascades.[3–8] Clinical studies have correlated increased *VEGF* mRNA and protein levels with tumor progression, leading to poorer prognosis and post-operative outcome in both NSLC and small cell lung cancer.[9–12] Binding of VEGF to its receptor stimulates the downstream kinases, ERK and AKT, driving proliferation, angiogenesis, cell invasion/migration, and cell survival, processes which are critical for lung tumor survival, growth, and metastasis.[13] Thus, reduction of *VEGF* expression could reasonably be expected to attenuate tumor growth and to represent a potential anti-cancer approach.

The promoters of many cancer-related genes, including *VEGF*, contain Guanine-rich DNA sequences which can form quadruplex (four-stranded) DNA. Almost all of these putative quadruplex-forming sequences are located within nuclease hypersensitive regions, suggesting that they likely play important regulatory roles, most commonly as gene silencers. On the other hand, tumor suppressor promoters are less likely to contain quadruplex-forming sequences.[14] Recent work has documented intracellular quadruplex formation [15, 16] and shown that quadruplex structures “mark” regulatory chromatin.[17] The *VEGF* quadruplex-forming sequence (VEGFq) is a 36bp G-C-rich region of the *VEGF* promoter (-85 to -50) which is essential for basal or inducible *VEGF* transcription. Its negative regulatory role in transcription has been demonstrated in vitro by the marked decrease of *VEGF* expression in the presence of quadruplex stabilizing agents.[18, 19]

The ability of oligonucleotides encoding genomic G-quadruplex forming sequences to specifically inhibit gene expression was initially shown in the response of leukemic cells to treatment with an oligonucleotide encoding the genomic c-MYC quadruplex-forming sequence (Pu27). Pu27 induced growth arrest and cell death in a variety of leukemic cell lines by oncosis through a mechanism involving inhibition of *c-MYC* mRNA and protein expression [20]. More recent work has demonstrated sequence-specific binding of the G-rich Pu27 oligonucleotide with the C-rich strand of its genomic target sequence, documenting strand invasion[21].

The random sequence G-rich quadruplex-forming oligonucleotide, AS1411, has been used as a therapeutic agent showing impressive anti-proliferative activity against a wide range of cancer cells, while being virtually nontoxic to normal cells.[20, 22–24] In Phase I and II clinical trials, AS1411 demonstrated significant clinical activity with the almost complete absence of toxicity [25]. The clinical experience with AS1411 demonstrated that quadruplex-forming oligonucleotides circumvent many of the common problems with oligonucleotide therapies. These include rapid nuclease degradation in serum and intracellularly, poor uptake into cancer cells, and “off target” effects on normal cells. In contrast to antisense oligonucleotides, quadruplex-forming oligonucleotides are inherently stable in biological fluids and effectively and preferentially taken into cancer cells.

It has been proposed that the quadruplex-forming sequence upstream of the *VEGF* promoter (VEGFq) plays an important role in modulating *VEGF* expression. This study shows that an oligonucleotide (ODN) encoding VEGFq inhibits proliferation and invasion of A549 NSCLC cells by decreasing VEGF production and consequently the signaling through ERK and AKT. We further demonstrate sequence-specific binding of the VEGFq encoding oligonucleotide to its double stranded target sequence indicating strand invasion, which results in stabilization of the quadruplex structure and transcription inhibition. This approach represents a new and clinically relevant strategy to inhibit *VEGF* expression and proliferation in tumor cells which overexpress *VEGF*.

## Materials and methods

### Cell culture

A549 (adenocarcinoma human alveolar basal epithelial cells), H1299 (lung adenocarcinoma), H1944 (lung adenocarcinoma), H3255 (lung adenocarcinoma), Calu-1 (epidermoid lung carcinoma) and as control nontransformed cells Hs27 (human fibroblast cells) and HPLD (immortalized human bronchiolar epithelial cells) (ATCC, USA) were maintained in DMEM media supplemented with 10% FBS and 100U penicillin/streptomycin at 5%CO2 and 37°C. Synthesized oligonucleotides (Oligos Etc.) were dissolved in RNAse/DNAse free ultrapure DH2O (Invitrogen) to a stock concentration of 500μM and boiled at 95°C for 5min. Cells in logarithmic growth phase were plated and treated with increasing concentrations of oligonucleotides VEGFq (5’-GGGGCGGGCCGGGGGCGGGG-3’) or the respective mutant control ODN (MutVEGF) (5’-G**TA**GCG**A**GCCG**TA**GGCG**A**G**T**-3) for various time intervals and used for subsequent biochemical analysis.

### Circular dichroism spectroscopy

VEGFq and MutVEGF oligonucleotides were annealed by boiling for five minutes in physiological buffer containing 20mM KH2PO4 dibasic, 120mM KCl, 5mM MgCl2 and slow cooled to room temperature. Annealed ODNs were dissolved in the same buffer at 5μM, a concentration which gives an absorbance of 0.800 at 260nm. Spectra were recorded on a Jasco-810 spectropolarimeter (Jasco, Easton, MD), using a quartz cell of 1 mm optical path length, an instrument scanning speed of: 200 nm/min, response time of 2 sec, and over a wavelength range of 340 nm to 220 nm. The spectra are representative of 3 average scans taken at 25°C and were baseline corrected for signal contributions due to buffer. CD data were normalized to strand concentration and are expressed in Molar Ellipticity [θ] (deg x cm^2^ x decimole^-1^).

### Electrophoretic mobility shift assay (EMSA)

PAGE purified duplexed VEGF target (84bp) was obtained from IDT and resuspended (100μM) in nuclease free water. Duplex DNA (double strand) VEGF target was incubated with 1x10^5^ cpm’s of ^32^P-labeled Target sequence were annealed in 1X TNE buffer (10mM Tris pH8.0, 90mM NaCl, 1mM EDTA) overnight at room temperature. The duplexed DNA was separated and purified from a 2% agarose gel. The product was incubated with 1x10^6^ cpm’s of ^32^P-labeled VEGFq in 20mM HEPES (pH 7.9), 25mM KCL, 2mM MgCl_2_, 0.1mM EDTA, 0.2mM DTT, 2mM spermidine and 10% glycerol for 15 min at 37°C. For competition, increasing concentration of “cold” VEGFq were added before the ^32^P-labeled VEGFq and incubated at 37°C for 15 minutes. Following incubation, an equal volume of glycerol dye (2X) was added. Half of the sample was electrophoresed on a 5% native polyacrylamide/TBE gel, fixed (10% methanol,10% acetic acid) for 15 minutes. An autoradiograph was obtained after exposure of film overnight at -80°C. EMSA for the binding with the single strands target, C-rich and G-rich covering 70bp of the same target (IDT) was performed by mixing 1.8μM of the target sequence with or without VEGFq at 3μM in SSC buffer. The mixes were denatured for 5 min in boiling water and annealed overnight at room temperature. Binding was analyzed by electrophoresis in denaturing polyacrylamide gel (12% polyacrylamide/8M Urea) using 2.5μl of the samples mixed with 2X glycerol dye before loading on the gel. A 10bp ladder (Invitrogen) was denatured and run in the 1^st^ well for size reference. At the end of the electrophoresis the gel was stained with SYBR Gold (Molecular Probes) and the bands visualized and photographed in Molecular Imager (Pharos FX Plus, Bio-Rad)

### MTT assay

Changes in cell proliferation of lung cancer cell lines and non-transformed fibroblasts in response to VEGFq, MutVEGF, or recombinant VEGF were assessed by 3- (4,5-dimethylthiazol-2yl)-2,5-diphenyltetrazolium bromide (MTT) assay. Cells were plated in a 96-well plate (1X103 cells/well) and incubated for 24 hours before they were treated with 1–15μM VEGFq or MutVEGF for 24 h-6 days. In some experiments, to test the reversibility of VEGFq, recombinant VEGF protein (1–50 ng/ml) was added to wells previously treated with VEGFq for 72 h. For all experiments, MTT reagent (100μ g/ml PBS) was added at the designated time and cells were incubated at 37°C and 5% CO2. After 4 h, cells were lysed and the formazan product was detected at 570nm on a spectrometer plate reader.

### Analysis of VEGFq and MutVEGF uptake

Cellular uptake of VEGFq or MutVEGF was analyzed by FACS and confocal microscope image analysis. A549 or Hs27 cells were incubated with 10μM of FITC-labeled VEGFq or MutVEGF for 1, 24, or 72 h, washed with PBS, and analyzed by FACS analysis. For confocal microscopy, Hs27 or A549 cells were treated for 72 h with FITC-labeled VEGFq or MutVEGF. After fixing cells in 4% paraformaldehyde for 20 min. and washing with PBS, cells were stained with DAPI (4’,6’-diamidino-2-phenylindole) nuclear stain and mounted with Prolong Gold (Invitrogen) anti-fade reagent. Cells were visualized by confocal microscopy with an Olympus Fluoview FV500 laser scanning microscope.

### Cell cycle analysis

A549 or Hs27 cells treated for 72 h with VEGFq or MutVEGF (10μM) were collected and washed in PBS. Cells were lysed with trypsin in spermine teterahydrochloride detergent buffer, and isolated nuclei were stained with propidium iodide (CycleTest Plus DNA Reagent, BD Biosciences) and analyzed by FACS analysis.

### Serum and intracellular stability of VEGFq

VEGFq and MutVEGF sequences were radiolabeled using [γ-32P]-dATP with T4 polynucleotide kinase (Life Technologies) and incubated in DMEM medium with 10% FBS at 37°C or in the presence of A549 S100 (cytoplasmic) extract for 0–72 h. Cold VEGFq or MutVEGF were added to give a final ODN concentration of 10 μM. At each time point, an equal volume of formamide dye was added and samples were quick frozen (ethanol bath) and stored at -80°C. After heating in 98% formamide buffer at 65°C, ODNs were run on a 12% denaturing gel and analyzed by autoradiography.

### Western blot analysis

Equal quantities of total cell lysates were separated by 4–15% SDS-Tris PAGE and electroblotted onto PVDF membranes. The membranes were blocked in 5% milk and incubated overnight at 4 C with a VEGF, ERK 1/2, p-ERK 1/2, AKT 1/2/3, or p-AKT 1/2/3antibodies (Santa Cruz Biotechnology). After washing, the membranes were incubated with a horseradish peroxidase (HRP)-conjugated secondary antibody. Proteins were visualized by standard chemiluminescence (ECL) methods (GE Healthcare). Equal loading of proteins was verified by probing the membrane with a mouse monoclonal anti-β-actin primary antibody (Santa Cruz Biotechnology).

### Boyden chamber invasion/migration assay

The Boyden chamber assay was used to assess the metastatic potential of A549 cells in response to VEGFq or MutVEGF. A549 cells pretreated with 10 μ M VEGFq or MutVEGF for 72 h, detached with TripleE (Life Technologies) and seeded (5x104/ml) in serum-free medium in the top side of the chamber containing an 8μm pore polycarbonate filter coated with Matrigel or in a control chamber without Matrigel (BD Biosciences). The chambers were placed in a 24 well plate with medium containing 10% FBS as a chemoattractant. VEGFq or MutVEGF (10μM) were added into the serum-free medium in the top chamber and the cells were incubated for 24h at 37°C. At the end of the incubation, cells on the upper surface of the membrane were carefully removed with a cotton swab and the cells that had invaded across the Matrigel to the lower surface of the membrane were fixed with 100% methanol and stained with crystal violet stain (Sigma-Aldrich) for 2 min. Excess stain was removed by washing in dH2O before drying overnight at room temperature. Membranes were then imaged using an Olympus microscope at 20X magnification. Six fields were photographed from each insert for quantification and analysis. Percent invasion was calculated by taking each Matrigel cell count and dividing by the respective control count (chamber without Matrigel). The invasion index was then calculated by dividing the treatment percent invasion by the untreated percent invasion.

### RT-PCR analysis of nuclear run-on RNA

Changes in newly synthesized mRNA (nuclear run-on RNA) were evaluated to more directly examine the effect of VEGFq on *VEGF* transcription, since the total level of mRNA is affected by both the rate of newly synthesized mRNA and mRNA stability. A549 cells (15x106) were treated with VEGFq or MutVEGF (10uM) for 72 h, collected by scraping, and washed with PBS. Nuclei were prepared and used in an RNA synthesis reaction containing biotin-16-UTP (Epicenter Biotechnologies) as previously described [23][15]. After the reaction was stopped with CaCl2 and RNAse-free DNAse I, total RNA was extracted from untreated cells or cells treated with VEGFq or MutVEGF using TRIzol (Invitrogen) according to manufacturer’s instructions. Nuclear run-on RNA (newly synthesized RNA) was isolated from total RNA using Dynabeads M-280 (Life Technologies) as previously described.[26] After the beads were washed once with 15% formamide (in 2X SSC) and twice with 2X SSC, they were resuspended in DEPC water and cDNA was prepared as previously described.[27] Primers were designed using Primer Express (Applied Biosystems): *VEGF* forward primer 5’-AGGCCAGCACATAGGAGAGATG-3’ and reverse primer 5’-AGGCCCACAGGGA TTTTCTT-3’ *ACTB* forward primer 5‘-TGCCGACAGGATGCAGAAG-3‘ and reverse primer 5‘- CTCAGGAGGAGCAATGATCTTGA-3’.Reactions were diluted 1:2 with SYBR Green I Master Mix (Applied Biosystems) and RT-PCR was performed for a uniform amount of cDNA using the Fast 7500 System (Applied Biosystems). A no template control reaction was run for each gene to control for DNA contamination of RNA extracts. *ACTB* was used to normalize the loading control. A dissociation curve was performed to provide evidence for a single reaction product. Expression of *VEGF* mRNA in VEGFq and MutVEGF-treated cells was compared to untreated cells collected at the same time point to calculate an expression ratio.

### LC3B expression

A549 cells treated with VEGFq or MutVEGF were fixed in 4% paraformaldehyde, washed with PBS, permeabilized (0.2% Triton) at room temperature, and blocked with 5% goat serum. After incubating with a rabbit anti-LC3B primary antibody (Life Technologies) in 1% goat serum overnight at 4°C, the cells were washed and incubated with an Alexa Fluor 488 anti-rabbit secondary antibody and analyzed by FACS. A control was performed under the same aforementioned conditions without primary antibody to demonstrate specificity.

### Electron microscopy

A549 cells were grown on ACLAR cover slips and treated with 10μM VEGFq or MutVEGF for 96 h. The cover slips were washed in cacodylate buffer, and immediately fixed in 3% glutaraldehyde in cacodylate buffer pH 7.4 overnight at 4°C. After post-fixation in 1% OsO4 in cacodylate buffer, the cover slips were dehydrated in graded ethanol, embedded, and sections were cut (80μM) with an ultramicrotome. Sections were stained with saturated aqueous uranium acetate and lead citrate and viewed with a Philips CM12 electron microscope operating at 60 KV.

### Data analysis

All values represent mean±SEM. Differences between treatments were determined by a non-paired T-test (2-tailed) using Sigma Stat software 3.5. A probability level of p<0.05 was used to indicate statistical significance.

## Results

### VEGFq forms a parallel quadruplex in solution

**Fig 1:**
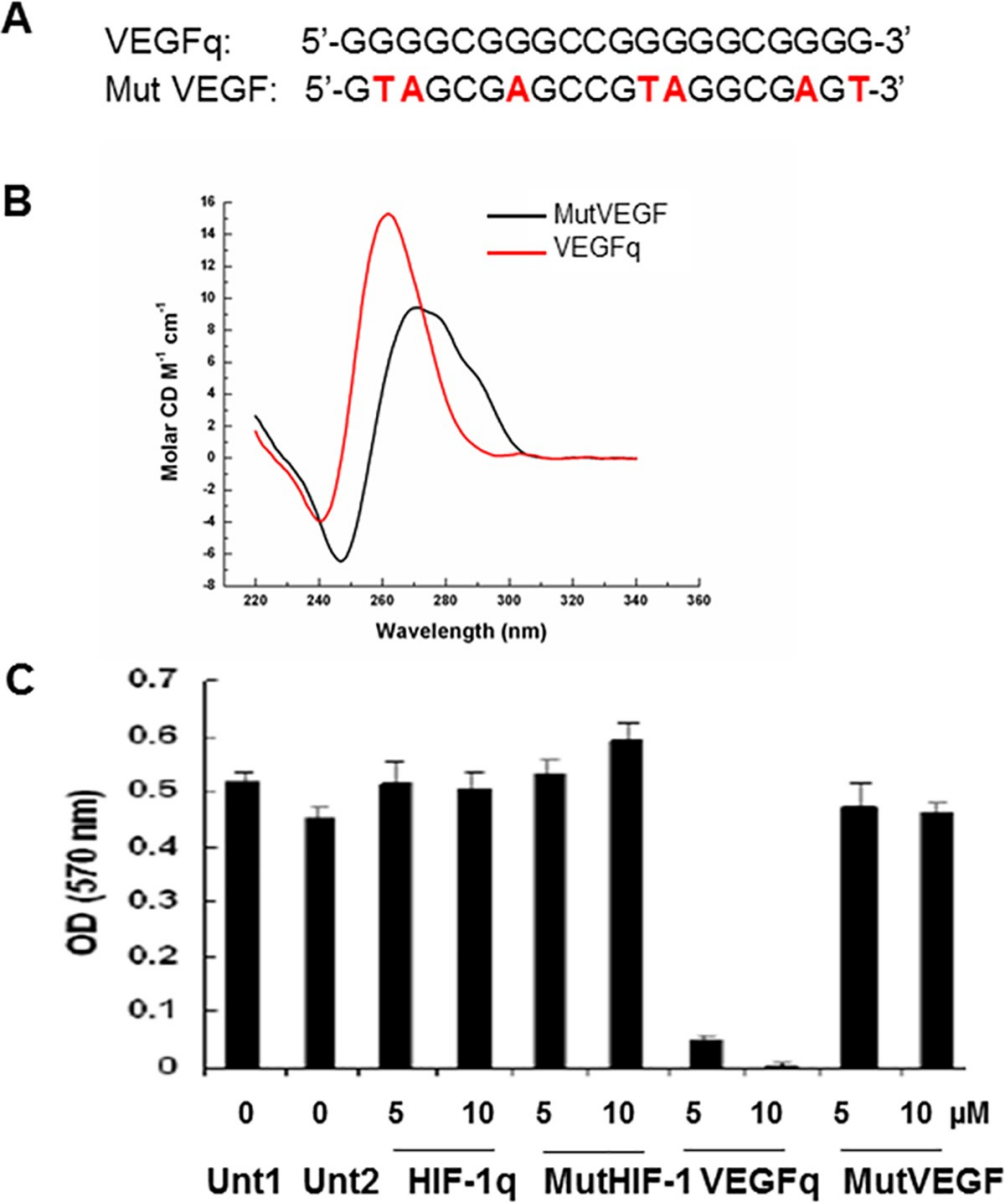
VEGFq forms a quadruplex structure in physiological solution and inhibits proliferation of lung cancer cells. (A) Nucleotide sequence of VEGFq and MutVEGF oligonucleotides. (B) Circular Dichrosim spectroscopy of VEGFq and MutVEGF oligonucleotides. Peak absorbance at 260 nm and trough absorbance at 240nm are indicative of G-quadruplex formation for VEGFq, compared to the MutVEGF sequence, which did not form a quadruplex. (C) Dose response to VEGFq or MutVEGF in A549 cells after 144 h by MTT assay. Controls include untreated cells or cells treated with a quadruplex-forming oligonucleotide targeted to the *HIF-1α* promoter (HIF-1αq) and a mutated HIF-1α (MutHIF-1α) oligonucleotide which did not form quadruplex.

The oligonucleotide encoding the genomic *VEGF* quadruplex-forming sequence used for these experiments is: 5’–GGGGCGGGCCGGGGGCGGGG-3’ (VEGFq) which encodes the 20bp quadruplex–forming sequence of the VEGF promoter. We also utilized a control (nonquadruplex-forming) oligonucleotide: 5’-G**TA**GCG**A**GCCG**TA**GGCG**A**G**T**-3 (MutVEGF) whose sequences had been altered to abrogate quadruplex formation (Fig 1A). Circular dichroism spectroscopy was used to characterize the secondary structure of VEGFq and MutVEGF. The VEGFq CD spectrum was characteristic of a parallel quadruplex in physiological solution, with a peak absorbance at 260 nm and a trough absorbance at 240 nm (Fig 1B). This confirms stable quadruplex formation by this oligonucleotide. MutVEGF, which was used as a control in these experiments, did not form a stable parallel quadruplex structure in solution. This lack of quadruplex-formation was predicted based on the sequence changes. This confirms the appropriateness of MutVEGF as a “nonquadruplex-forming” control for subsequent experiments.

### VEGFq inhibits proliferation of lung cancer cells

Initial experiments tested the ability of the VEGFq oligonucleotide to inhibit the growth of A549 NSCLC cells which overexpress *VEGF*. A549 cells were treated with VEGFq and MutVEGFq at concentrations of 5 and 10μM for 144 hours (Fig 1C). VEGFq had a dramatic effect on the growth of A549 cells, while MutVEGFq had very little effect. The A549 cells were also treated with a quadruplex-forming oligonucleotide targeted to the quadruplex-forming sequence of the *HIF-1α* promoter (HIF-1αq). Importantly, the quadruplex-forming oligonucleotide (documented to form quadruplex DNA by circular dichroism spectroscopy) targeted to the *HIF-1α* promoter did not impede proliferation of the A549 cells, indicating that the effect of VEGFq is not simply a toxic effect of quadruplex-forming oligonucleotides, but reflects a more specific effect.

**Fig 2:**
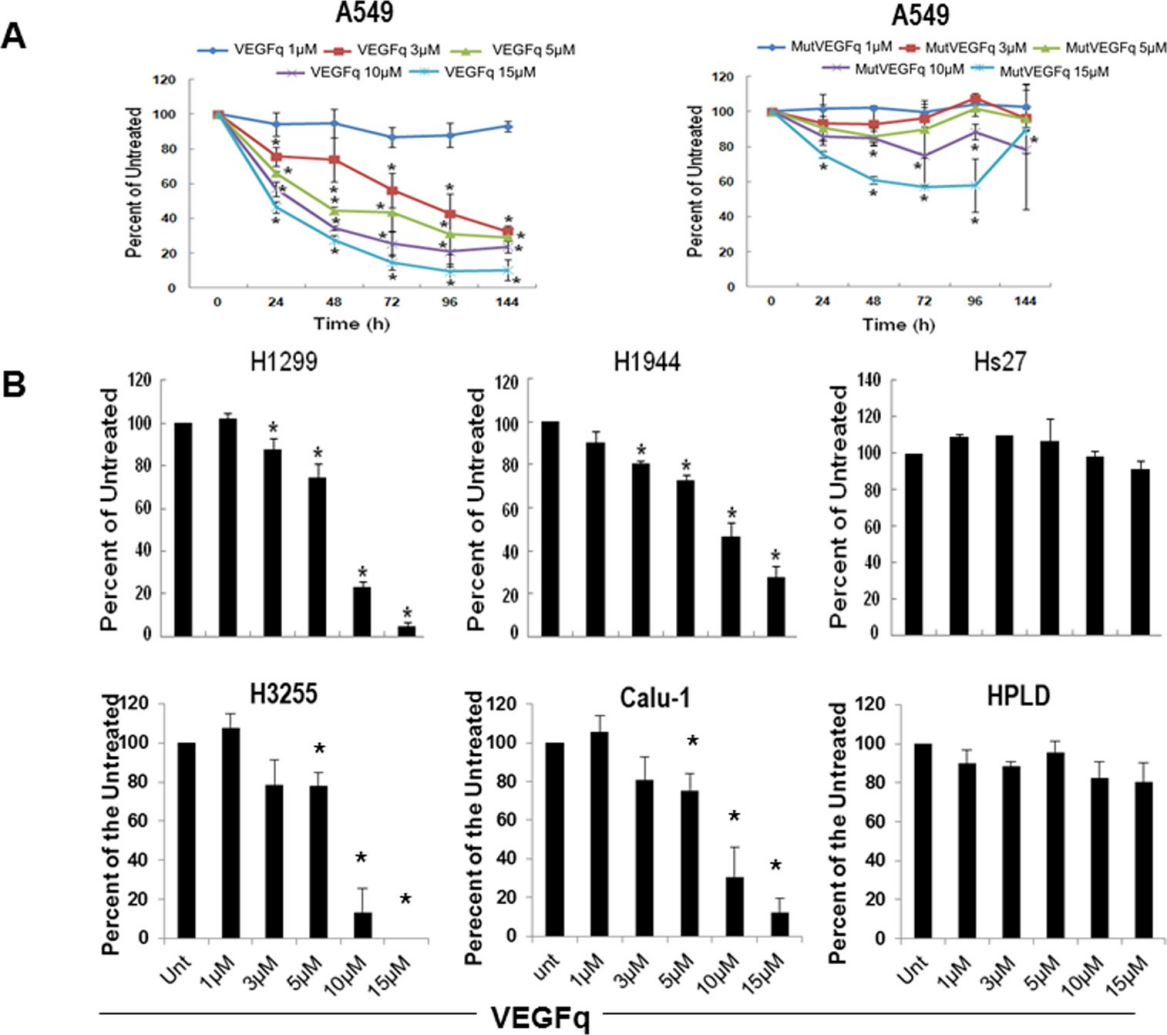
VEGFq inhibits cell proliferation of NSCLC cells but not non-transformed cells in a concentration and time dependent manner. Proliferation was measured by the MTT assay. (A) VEGFq, but not MutVEGFq inhibited the growth of A459 cells in a concentration and time dependent fashion. (B) VEGFq also inhibited the growth of the H1299 and H1944 NSCLC cell lines, H3255 squamous cell lung cancer cells, and the Calu-1 epidermoid carcinoma cell line, but had very little effect on the non-transformed foreskin fibroblast Hs27 cells and the lung epithelial cells, HPLD-1 measured at 144 h. Bars represent mean±SEM absorbance of three separate determinations. * indicates (p<0.05) compared to untreated cells.

Subsequent experiments characterized the time and dose dependence of the VEGFq effect on A549 cells. Cultures were treated with 1–15μM VEGFq for 24–144 h demonstrating a dose and time-dependent decrease in proliferation, culminating in 80% inhibition with 10μM and 90% inhibition with 15μM VEGFq after 6 days (IC50 of less than 5μM) compared to untreated cells **(**Fig 2A). On the other hand, MutVEGFq caused relatively little inhibition of proliferation of A549 cells. The effect of VEGFq on A549 cells was consistent with that seen with other NSCLC cell lines, as shown in Fig 2B. Both the H1299 and H1944 non-small cell lung cancer cell lines demonstrated dose dependent growth inhibition when exposed to VEGFq. Treatment of H3255 squamous cell lung cancer and the Calu-1 (epidermoid carcinoma) cell lines demonstrated an equivalent degree of growth inhibition (Fig 2B). Importantly, treatment of Hs27 non-transformed human fibroblast cells with VEGFq caused no significant change in cell proliferation when compared to A549 cells treated in parallel. Similar results were seen with the human peripheral lung airway cell line (HPLD-1) (Fig 2B). These results suggest that growth inhibition by VEGFq is specific for transformed cells compared to non-transformed cells.

**Fig 3:**
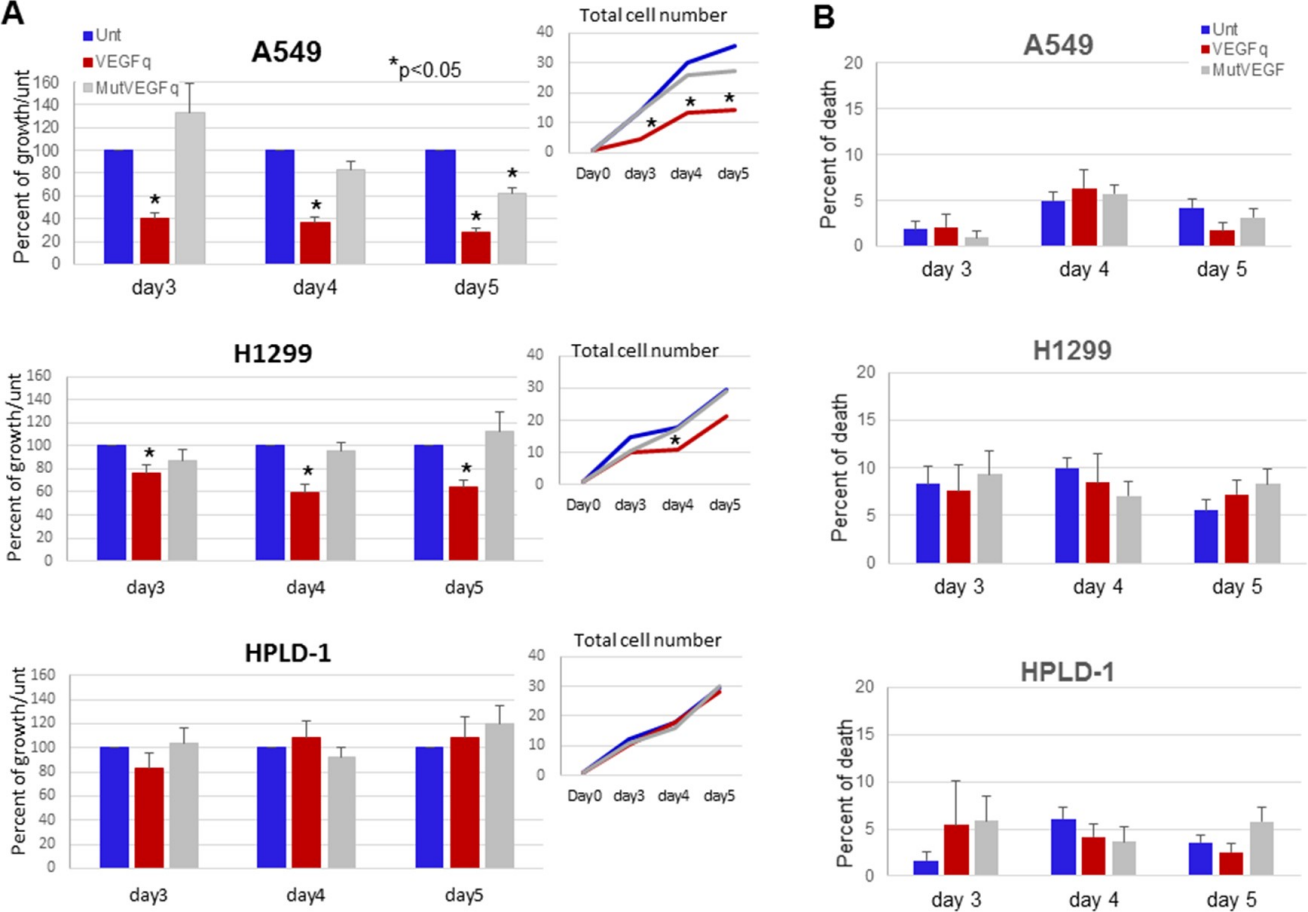
VEGFq inhibit cell growth but does not increase cell death in Lung cancer cells. A549, H1299 and HPLD-1 were exposed to 10μM of VEGFq or MutVEGFq for 3, 4 or 5 days and then counted in Trypan blue. (A) Percent of cell growth compared to untreated, inset: total number of cells. (B) Percentage of cell death. Data represent the averaged cell counts for 3 separate experiments realized in duplicate +/- SEM, * for p<0.05.

To determine whether the decrease in cell proliferation was due to cell death, A549 (overexpressing VEGF), H1299 (moderately expressing VEGF) and the control non-transformed HPLD-1 were exposed to VEGFq or MutVEGFq at 10μM for 3, 4 or 5 days and counted using the dye exclusion assay (Trypan Blue). As shown in Fig 3A, A549 and H1299 cells exposed to VEGFq had a significant reduction in proliferation, while MutVEGFq had minimal effect and only at 5days. The cell number was significantly decreased for A549 and H1299 treated with VEGFq but not for HPLD-1 (Fig 3A insert). In addition, there was no significant increase in cell death in the treated cells compared to untreated (Fig 3B). This data confirm the result obtained with the MTT experiments and demonstrated that the exposure to oligonucleotide targeting VEGF reduce lung cancer cells growth.

### Recombinant VEGF protein partially rescues lung cancer cells from the growth inhibitory effects of VEGFq

**Fig 4:**
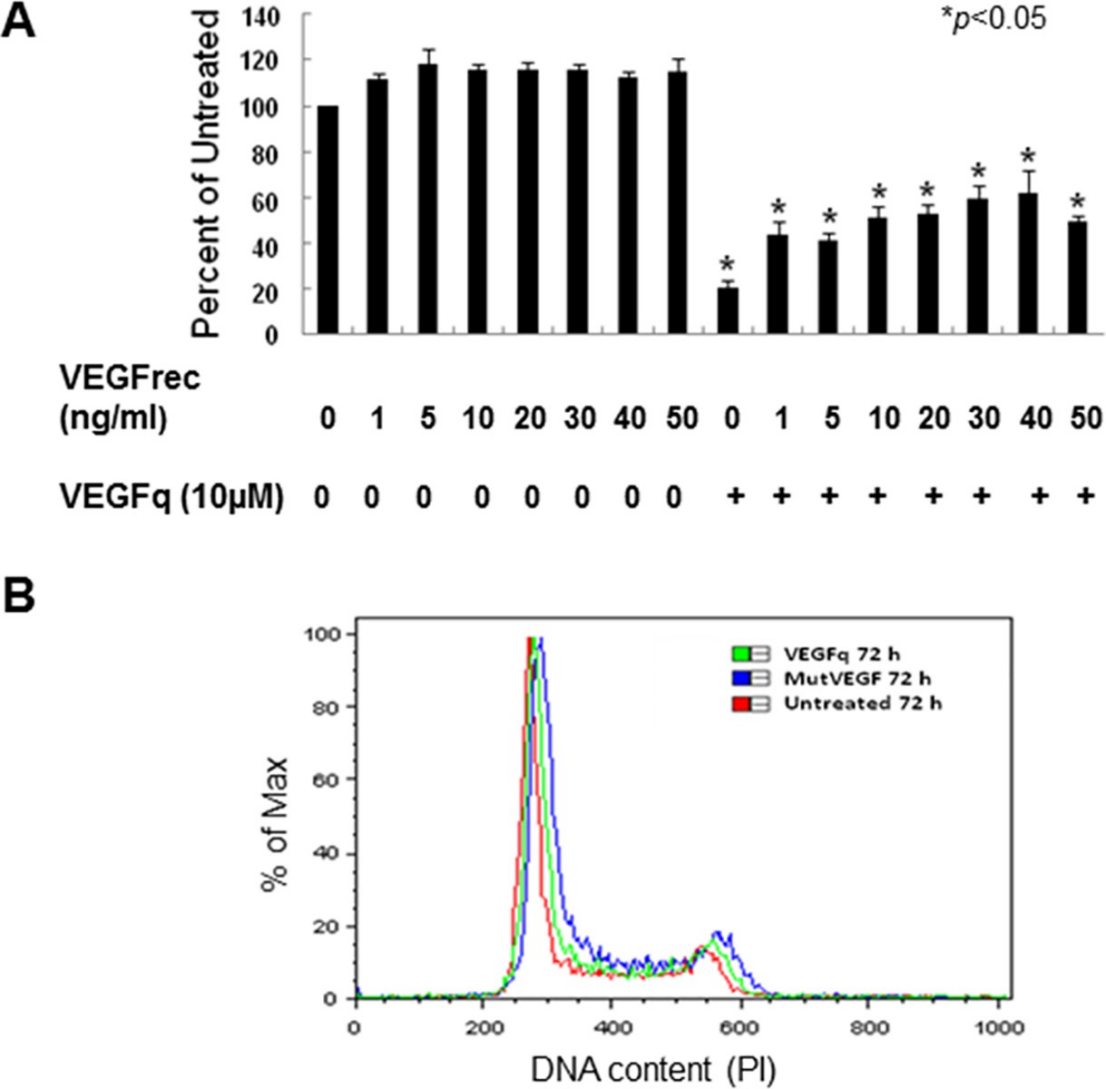
VEGFq induced inhibition of cell growth can be partially rescued by recombinant VEGF and the absence of cell cycle changes. (A) The effect of treatment of A549 cells with recombinant VEGF (1–50 ng/ml) with and without VEGFq pretreatment on cell proliferation after 144h by MTT assay. Bars represent mean±SEM absorbance of three separate determinations. * indicates (p<0.05) compared to untreated cells. Results show that addition of recombinant VEGF to VEGFq-treated cells significantly diminishes the growth inhibitory effect of VEGFq treatment. (B) Flow cytometry analysis of cell cycle for A549 cells treated with VEGFq and MutVEGF. No changes in the cell cycle were noted.

In order to determine if the growth inhibiting effects of VEGFq are caused by decreased *VEGF* expression, it was important to determine whether these effects can be reversed through addition of recombinant VEGF (VEGFrec) protein. Thus, A549 cells were treated with 10μM VEGFq for 4h, then VEGFrec was added and cell growth was analyzed by the MTT assay over six days (Fig 4A). Compared to cells treated with VEGFq alone, addition of recombinant VEGF restored the VEGFq-induced decrease in cell proliferation by as much as 40**%** after 6 days. Although an increase in cell proliferation in response to recombinant VEGF occurred, it did not increase with escalating concentrations of recombinant VEGF, suggesting receptor saturation at lower levels. These results suggest that VEGFq-mediated inhibition of cell proliferation likely depends on inhibition of *VEGF* expression.

### VEGFq and MutVEGF do not affect the cell cycle

Previous studies of G-quadruplex-forming oligonucleotides have demonstrated dramatic alterations of cell cycle parameters in responsive cells. Thus it was important to determine if treatment of A549 cells with VEGFq or MutVEGF caused an overall change in the cell cycle. To this end, A549 cells were incubated with 10μM VEGFq or MutVEGF for 72 h and analyzed by flow cytometry for DNA content using PI incorporation. No significant change in the cell cycle occurred with VEGFq or MutVEGF treatment compared to untreated cells (Fig 4B). This is a clear distinction from the results seen in cells treated with the random quadruplex-forming oligonucleotide, AS1411 [23, 25], or with the c-MYC targeted Pu27 [20]. This difference most likely reflects the gene-specificity of VEGFq and the different pathway involved in the growth inhibition induced by VEGFq.

### Cellular uptake of VEGFq and MutVEGF

**Fig 5:**
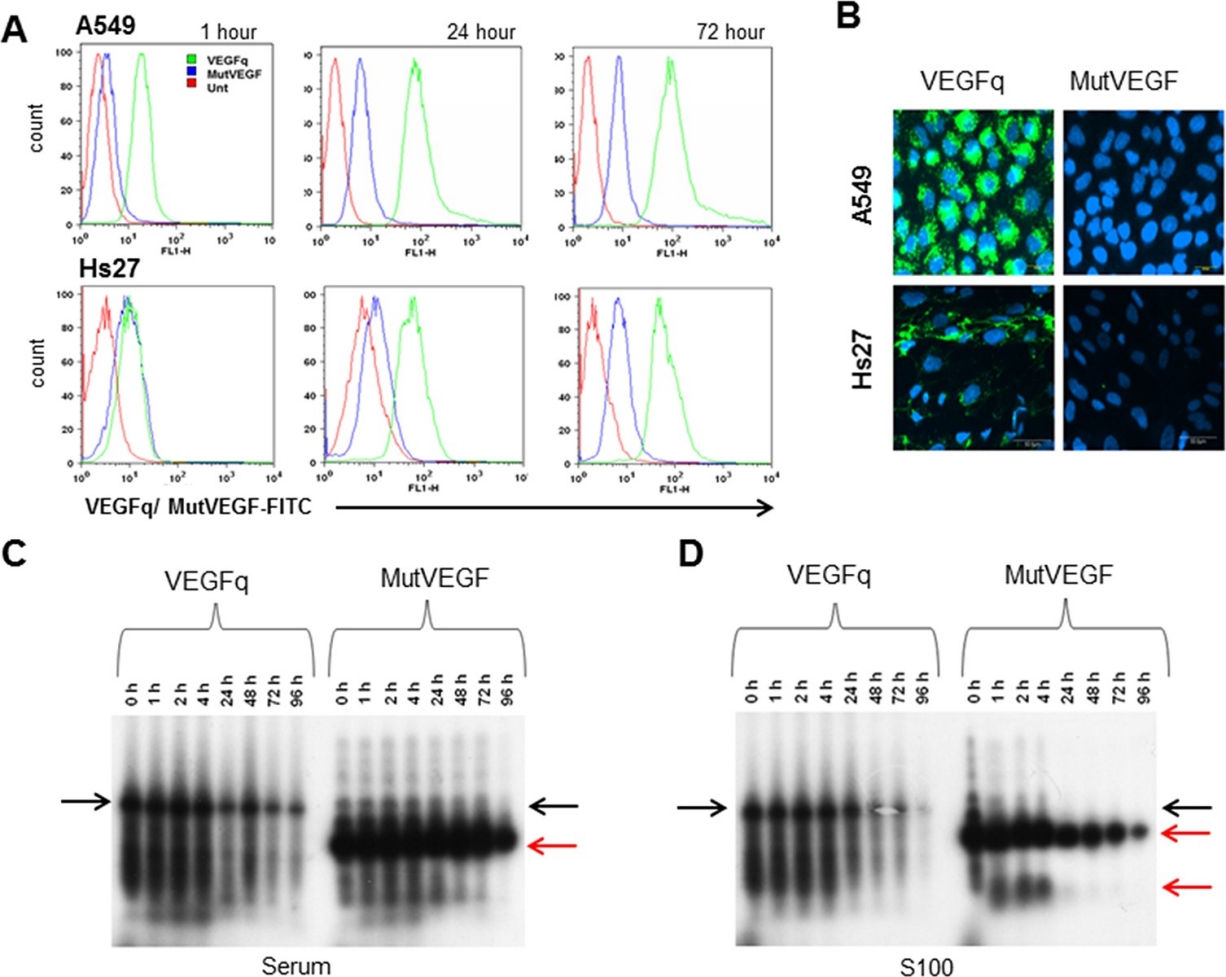
Cellular uptake and stability of VEGFq or MutVEGF in A549 cells. (A) Uptake of the FITC-labeled VEGFq or MutVEGF oligonucleotides (10μ M) in A549 and Hs27 after 1h, 24h, or 72h determined by FACS analysis (B). Confocal microscopy analysis of A549 or non-transformed fibroblast Hs27 cells treated with 10μM FITC-labeled VEGFq or MutVEGF after 72hr. In microscopic analysis, cell nuclei were stained with DAPI. Note significant uptake of VEGFq compared to MutVEGF in A549 and Hs27 cells. (C-D) 32P-labeled VEGFq and MutVEGF sequences were incubated in DMEM media at 37°C with 10% FBS (C) or A549 S100 cell extract (D) for 0–72 h. Black arrows indicate intact ODNs and red arrows the denatured products. Greater serum and intracellular stability occurred with VEGFq compared to MutVEGF. Note immediate degradation of MutVEGF into a secondary product.

An important factor in oligonucleotide efficacy is cellular uptake. Unmodified phosphodiester oligonucleotides, such as VEGFq, are often unstable and only poorly internalized by tumor cells. We characterized the uptake of VEGFq and MutVEGF using fluorescently labelled oligonucleotides. FITC-VEGFq was immediately taken into A549 cells after 1 h, with enhanced uptake after 24 and 72 h (Fig 5A). Significantly less FITC-MutVEGF appeared to be taken into cells at all time points. Confocal microscopy at 72h confirmed these results, showing both cytoplasmic and nuclear localization of FITC-VEGFq and almost no uptake of MutVEGF at the same intensity settings (Fig 5B). Similarly, non-transformed Hs27 cells also took up FITC-VEGFq after 72 h, with greater uptake over time, which appeared more largely localized in the cytoplasm (Fig 5A). These results suggest that VEGFq is more effectively internalized by A549 cells than is MutVEGF and is largely localized in the nucleus and cytoplasm of these cells. Uptake of VEGFq into Hs27 cells suggest that the lack of anti-proliferative effect of VEGFq on non-transformed cells is not caused by its inability to be taken into cells, but more likely reflects the fact that these cells are not dependent on VEGF for their growth characteristics.

### VEFGq is stable in serum and in cytoplasmic extract

The biologic activity of ODNs is often limited by their susceptibility to nuclease degradation. This is particularly true of unmodified, phosphodiester oligonucleotides, such as VEGFq. Thus, we characterized, the stability of VEGFq and MutVEGF both in serum and in a cytoplasmic extract. As shown in Fig 5C and 5D, 32P-labeled VEGFq was inherently stable in serum and in the presence of A549 cytoplasmic extracts (S100), while MutVEGF was almost immediately degraded into a smaller product in the presence of serum (Fig 4C). This remarkable superior stability of VEGFq is likely related to the inherent nuclease resistance of G-quadruplex DNA, that was confirmed by the demonstration of markedly decreased stability of the non-quadruplex-forming MutVEGFq. This observation is extremely important in characterizing the potential therapeutic applications of VEGFq.

### VEGFq induces autophagic cell death in NSCLC cells

**Fig 6:**
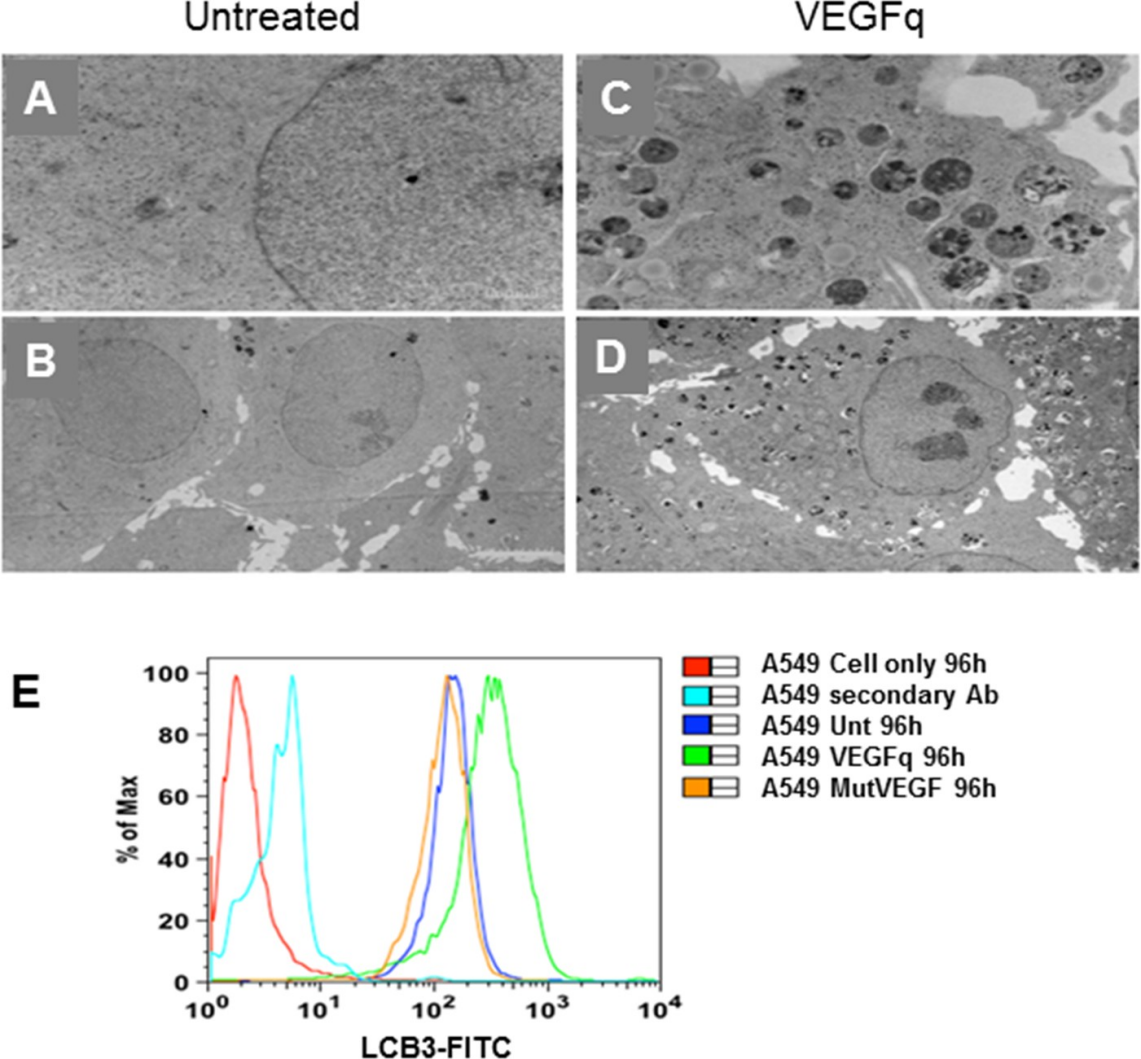
VEGFq induces authophagic cell death in NSCLC cells. Electron microscopy images of untreated A549 cells (A, B) and A549 cells treated with 10μM VEGFq for 96h (C, D). VEGFq treatment caused dense cytoplasmic accumulation of autophagic vacuoles compared to untreated cells. (original magnification 8800X-A, C; 3400X-B, D). (E) FACS analysis of LC3B staining (FITC) as an autophagosome marker, confirmed induction of autophagy. Treatment of A549 cells with VEGFq (green) for 96h increased LC3B expression compared to untreated (blue) and cells treated with MutVEGF (orange). Minimal staining was noted with secondary antibody only (teal), confirming specificity.

Previous work has shown dramatic morphologic changes induced by G-quadruplex-forming oligonucleotides[20], including increase in size and granularity of the treated cells[28]. Electron microscopic characterization of A549 cells treated with VEGFq for 96 h demonstrated dense cytoplasmic accumulation of autophagic vacuoles with significant engulfment of cellular components compared to untreated cells (Fig 6C and 6D). There was no evidence of a compromised cell membrane or ultrastructural damage (Fig 6A–6D). During the formation of autophagosomes, the microtubule associated protein 1A/1B-light chain 3 (LC3) becomes post-translationally modified by phosphatidyle ethanolamine and is incorporated into the membrane. To verify the autophagic nature of the vacuoles, we evaluated the presence of LC3B, which associates with the phagosomes and plays a critical role in autophagy (Fig 6E). After 96h of VEGFq treatment, there was an increase in LC3B staining compared to untreated cells or cells treated with MutVEGF. Cells stained only with secondary antibody showed minimal signal, demonstrating specificity of the primary antibody. This is consistent with an autophagic mechanism of cell death and, corresponds with the absence of VEGFq induced cell cycle changes, most likely reflecting a distinct (gene-specific) mechanism of action of VEGFq. In addition, VEGFq treatment for up to 96h did not cause a significant increase in Trypan Blue positive cells suggesting an absence of cell membrane permeability as reported in the Trypan blue dye exclusion assay (Fig 3B). This is quite consistent with autophagy as the mechanism of cell death induced by VEGFq.

### VEGFq inhibits cell invasion

**Fig 7:**
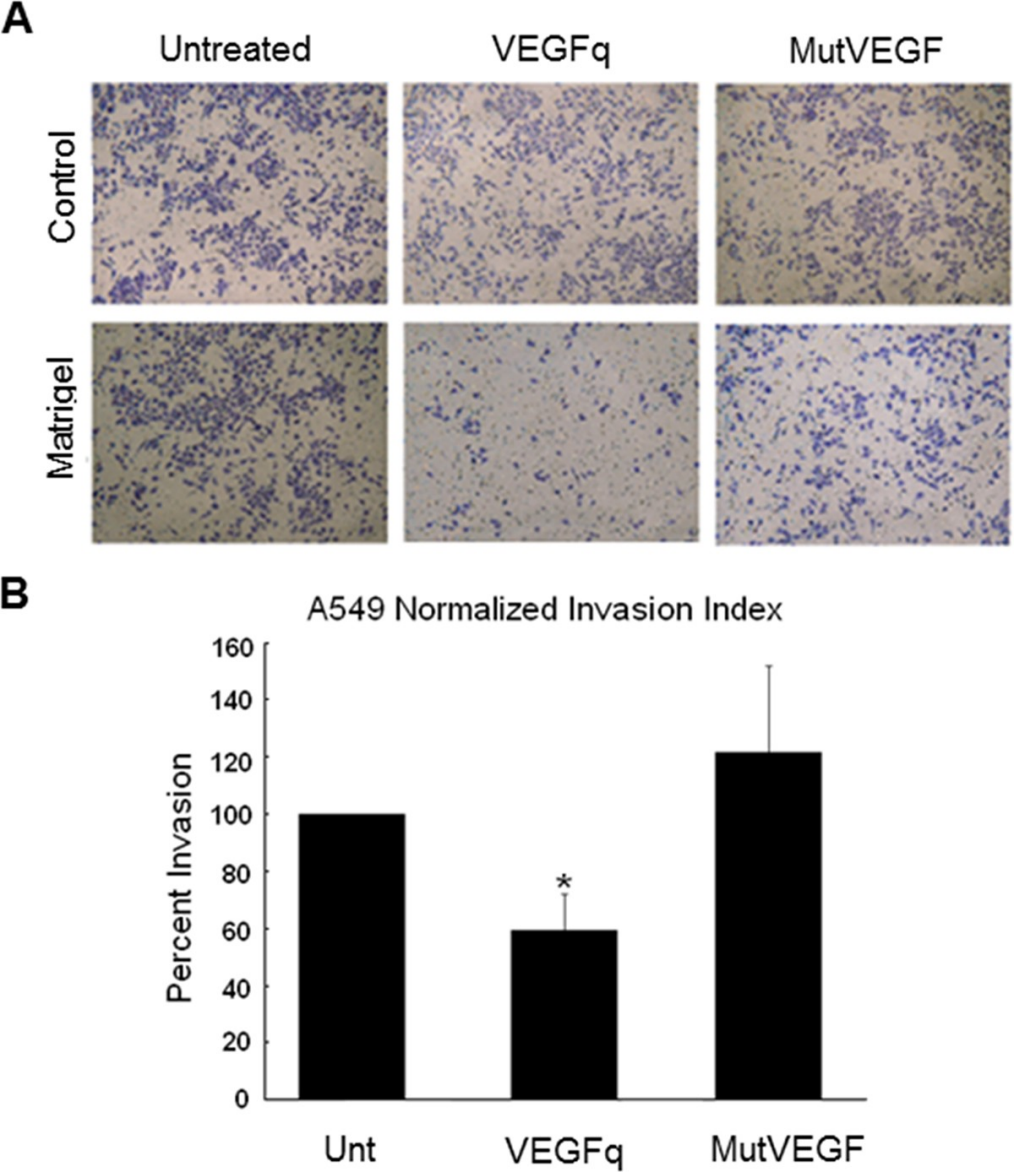
VEGFq inhibits A549 cell invasion. (A) Treatment of A549 cells with VEGFq (10 μM) significantly decreased cell invasion (Matrigel) after 24 h compared to untreated and cells treated with MutVEGF determined by Boyden chamber. No change in migration (control) was noted. (B) Invading cell counts were normalized to migrating cell counts to calculate a normalized invasion index. Bars represent mean±SEM absorbance of three separate determinations. * indicates (p<0.05) compared to untreated cells.

A major role of *VEGF* in transformed cells is to increase mobility and invasiveness. In order to determine whether VEGFq treatment alters *VEGF* expression in a way that affects cell invasion, A549 cells were treated with 10μM VEGFq for 24h, then evaluated for migration/invasion by Boyden chamber analysis. Compared to untreated control cells, VEGFq decreased A549 Matrigel cell invasion by 50%, while MutVEGF had no effect (Fig 7A and 7B). There were no significant changes in cell number of the control plates in response to VEGFq or MutVEGF treatment demonstrating little or no effect on cell migration (Fig 7A) suggesting that the decrease observed the invasion assay in VEGFq treated well is not due to a difference in cell number. This result is consistent with the expected decreased invasiveness seen with inhibition of *VEGF* expression and/or activity.

### VEGFq treatment decreases newly transcribed mRNA and intracellular VEGF protein levels

**Fig 8:**
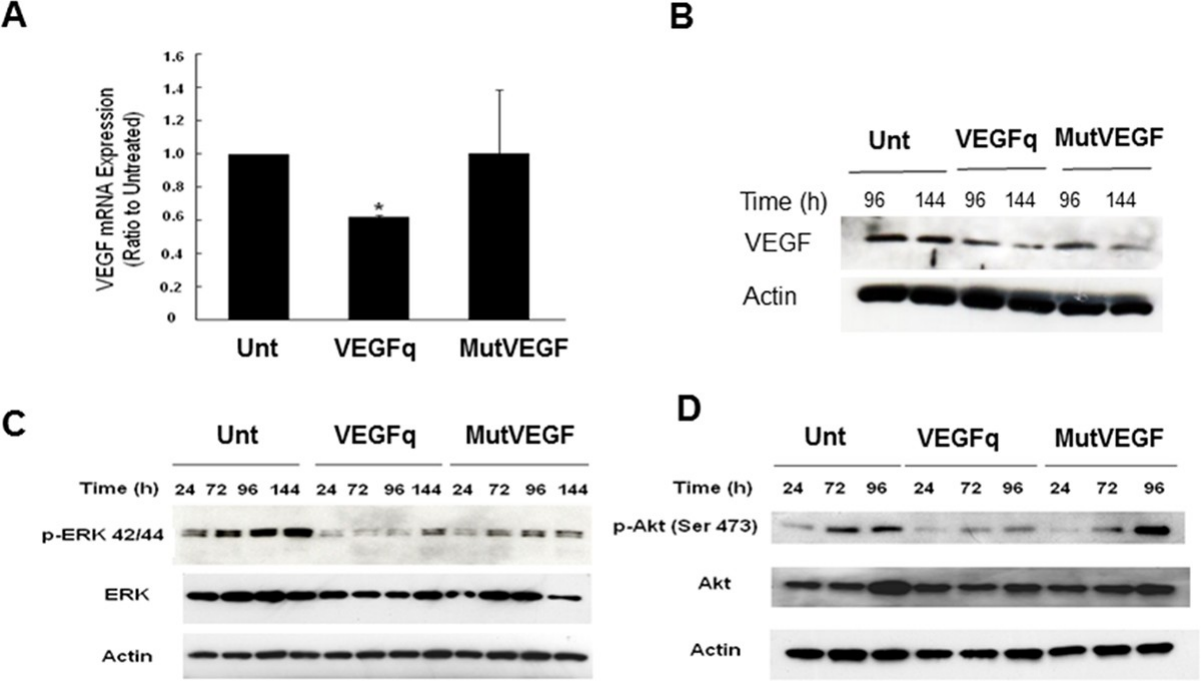
VEGFq treatment decreases newly transcribed *VEGF* mRNA and protein expression and downstream ERK1/2 and AKT mediated signaling. (A) Nuclear run-on *VEGF* mRNA after 72h and (B) VEGF protein expression after 96/144h exposure to 10μμM VEGFq or MutVEGF as determined by RT-PCR and Western blot analysis respectively. Note a 40% decrease in newly synthesized *VEGF* mRNA and a profound decrease in protein expression. Actin was used as the loading control for both RT-PCR and Western blot experiments. Bars represent mean±SEM absorbance of three separate determinations. * indicates (p<0.05) compared to untreated cells. Blots are a representation of three independent experiments. (C) Time course of ERK 1/2 / p-ERK 42/44 and (D) AKT / p-AKT Ser 473 protein expression after 24-144h of 10μM VEGFq or MutVEGF treatment as determined by Western analysis. Actin was used as the loading control. Note substantial loss of ERK 1/2 and AKT activity after VEGFq treatment. Blots are a representation of three independent experiments.

In order to better understand the mechanism by which VEGFq inhibits proliferation of NSCLC cells, we investigated its effect on run-on transcription of *VEGF*. VEGFq treatment of A549 cells decreased newly synthesized *VEGF* mRNA by 40% after 72h compared to untreated and cells treated with MutVEGF (Fig 8A). In general, VEGF protein expression paralleled *VEGF* mRNA levels. Although treatment of A549 cells with VEGFq did not decrease VEGF protein levels after 24 and 72h, there was a marked decrease in expression (< 60% of untreated) after 96h and 144h (Fig 8B). The delayed decrease in VEGF protein levels can be explained by the relative stability of *VEGF* mRNA and protein. MutVEGF treatment did not alter VEGF mRNA expression levels, consistent with the non-specifity of the mutated oligonucleotide, however, the protein level was decreased by 6 days. These results are consistent with a mechanism in which VEGFq inhibits transcription and, ultimately, translation of the *VEGF* gene, most likely by sequence-specific Watson Crick binding to the target sequence in the *VEGF* promoter.

### Effect of VEGFq on MAPK signaling

To further evaluate the mechanism of action of VEGFq and the consequences of VEGF downregulation on cell proliferation and invasion we evaluated VEGFq downstream pathways. The effect of VEGFq on the ERK and AKT pathways was investigated in A549 cells. Decreased cellular *VEGF* expression with VEGFq treatment corresponded with a small decrease in total ERK 1/2 levels and a marked decrease in the activated (phosphorylated) form of p-ERK 1/2 (p42/p44), beginning at 24 h thorough 144 h compared to untreated (Fig 8C). Although some effect was noted with MutVEGF treatment, VEGFq significantly decreased p-ERK 1/2 (p42/p44) at 72 and 96h compared to MutVEGF. As was the case of ERK, total AKT levels remained relatively constant after treatment with VEGFq. However, levels of the activated form, p-AKT was also significantly decreased after 72 and 96h by VEGFq (Fig 8D). MutVEGF had only a very weak effect on the AKT pathway. These results suggest that *VEGF* downregulation induced by VEGFq decreases downstream signaling through ERK and AKT, and is related to the inhibition of cellular proliferation and migration/invasion.

### Sequence specific binding of VEGFq to its double stranded target sequence

**Fig 9:**
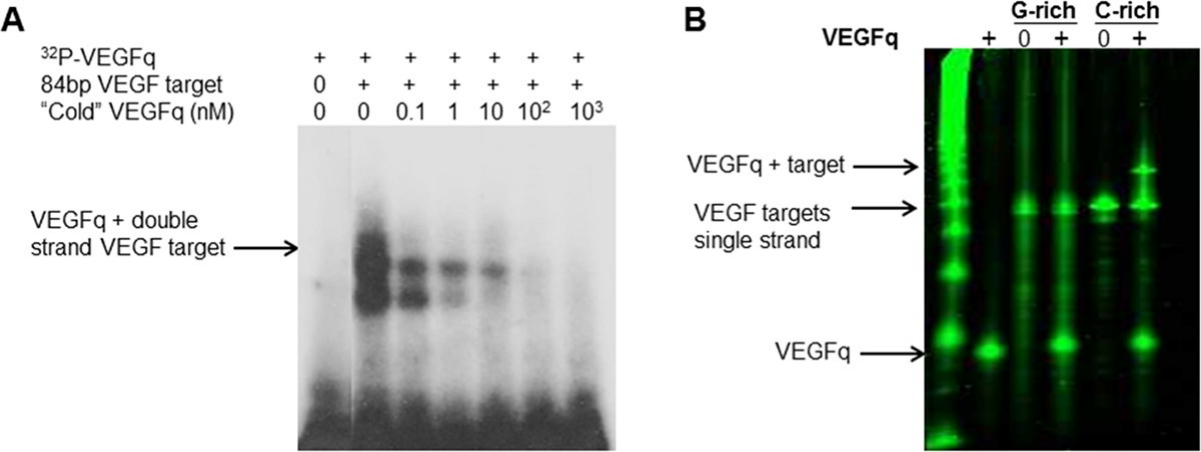
VEGFq binds to its double stranded target sequence by strand invasion. (A) Electrophoretic mobility shift assay (EMSA) of VEGFq binding to an 84bp duplex target sequence. Radiolabeled VEGFq oligonucleotide was added to an excess of target sequence. Two retarded bands were observed, both of which were abrogated by the addition of unlabeled VEGFq, indicating sequence specific binding. (B) EMSA representing the binding of VEGFq to the C-rich single strand of the target VEGF promoter sequence, as expected, no binding was noted to the G-rich strand suggesting the VEGFq binds to the C-rich strand by strand invasion, rather than to the G-rich strand by intermolecular G-quadruplex formation.

**Fig 10:**
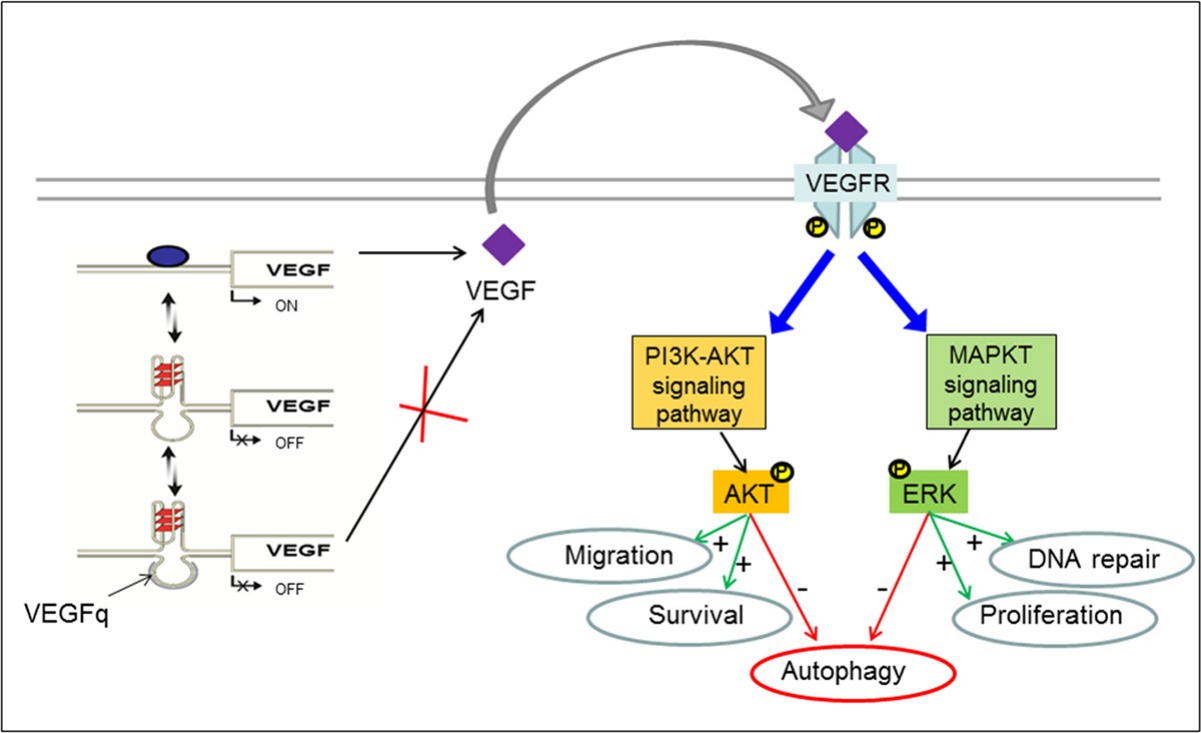
Cartoon depicting the “strand invasion model” of VEGFq action in NSCLC. VEGFq binds to the *VEGF* promoter and stabilize the G-quadruplex structure on the opposite stand, this results in downregulation of VEGF transcription. The reduced expression of VEGF failed to activate MAPK pathway, notably the activation of ERK and AKT that has been shown to inhibit autophagy (red arrows). Therefore, by altering the MAPK pathway, VEGFq inhibits proliferation and invasion and promotes autophagic cell death.

It has previously been shown that modified oligonucleotides (peptide-nucleic acids) can bind to quadruplex-forming regions by strand invasion.[29–32] More recently it has been shown that phosphodiester oligonucleotides encoding the G-rich strand of the c-MYC quadruplex forming sequence, Pu27, bind specifically to the complementary C-rich strand of the target sequence.[21] Strand invasion is sequence-specific because it involves Watson-Crick binding to the complementary strand. In quadruplex-forming sequences, it induces stabilization of the quadruplex structure, theoretically resulting in transcriptional inhibition. In these G-rich sequences, strand invasion by a G-rich oligonucleotide may be favored because the quadruplex structure favors strand separation. As shown in Fig 9A, radiolabeled VEGFq oligonucleotide was incubated with an 84 bp double stranded DNA fragment encoding the *VEGF* promoter segment which contains the VEGFq sequence. The resulting EMSA demonstrates specific binding of VEGFq to its double stranded target sequence. The two bands most likely represent distinct quadruplex structures. The binding is completely competed by increasing amounts of unlabeled oligonucleotide which abrogates both retarded bands. Furthermore, we demonstrate (Fig 9B) that VEGFq binds only to the C-rich strand of the target sequence, as expected and does not form an intermolecular quadruplex structure. This result indicates that VEGFq binds to its target sequence by strand invasion accounting for the gene-specific effects that we have documented. The binding of VEGFq to the C-rich strand of the genomic target sequence would be expected to stabilize the G-quadruplex structure of the G-rich strand, as shown in the cartoon in Fig 10.

## Discussion

The promoters of many cancer-related genes, including *VEGF*, contain sequences that are capable of forming intramolecular quadruplex (four-stranded) DNA structures [33]. These G-rich sequences are generally located within nuclease hypersensitivity regions which play important roles in regulating expression of their downstream genes. Using quadruplex-specific antibodies, Balasubrumanian has shown that quadruplex formation occurs in intact cells [15, 16] and that quadruplex structures are disproportionately represented in regulatory chromatin [17]. Previous work has suggested that the quadruplex-forming sequence within the *VEGF* promoter plays an important role in downregulation of basal and inducible *VEGF* transcription [8,19,24]. This is consistent with the transcriptional inhibitory activity of quadruplex-forming sequences in other promoters, including the c-MYC [34] and hTERT [35] promoters. In fact, mutations in the *hTERT* quadruplex-forming sequence are seen quite commonly in melanoma [36, 37] and glioblastoma, [38] and less commonly in other tumors, as well. These mutations have been shown to destabilize the *hTERT* promoter quadruplex structure and, presumably, abrogate the transcriptional inhibitory activity which occurs after quadruplex formation. [39]

Treatment of leukemic cells with oligonucleotides encoding the genomic c-MYC quadruplex-forming sequence, Pu27, induces growth arrest and cell death in several leukemic cell lines by inhibiting c-MYC expression.[20] This observation was the first report of selective cancer cell killing by a genomic DNA sequence. More recently, it has been shown that inhibition of c-MYC expression occurs via transcriptional inhibition caused by strand invasion of the *c-Myc* targeted oligonucleotide resulting in quadruplex stabilization.[21] Growth inhibition induced by the Pu27 oligonucleotide is preceded by very striking DNA damage.[28] Since *VEGF* is highly overexpressed in lung cancer and its overexpression is commonly associated with poor prognosis [40–43] we elected to study the effect of oligonucleotides encoding the genomic *VEGF* promoter quadruplex-forming sequence on NSCLC cells. Because of the unique stability and cellular uptake characteristics of quadruplex forming oligonucleotides, these experiments were conducted with unmodified phosphodiester oligonucleotides in the absence of any uptake enhancement strategy.

The data presented here clearly support the “gene-specific” inhibition of *VEGF* expression by the VEGFq oligonucleotide. The specific binding of VEGFq to its double stranded target sequence in the VEGF promoter (EMSA experiments) strongly supports Watson-Crick binding by strand invasion. Although this has been shown previously for peptide-nucleic acids,[31, 44] the ability of an unmodified phosphodiester oligonucleotide to bind specifically to the C-rich strand likely stabilizes the quadruplex structure of the G-rich strand, as indicated by the cartoon in Fig 10. Shifting the intracellular equilibrium from duplex DNA to the quadruplex form of the VEGF promoter would be expected to block transcription initiation of the *VEGF* promoter, resulting in inhibition of VEGF expression, in a manner consistent with the data which we present.

As was the case with previous experiments testing the effect of c-MYC targeted oligonucleotides, treatment with VEGFq addresses many of the significant biological limitations of oligonucleotide therapy including low toxicity to normal cells, abundant uptake into cancer cells in the absence of any uptake enhancement strategy, and remarkable stability in biological fluids. The results of this study also suggest probable gene specific inhibition by a genomic quadruplex-forming oligonucleotide, and supports strand-invasion as a unique strategy for abrogating *VEGF* expression in NSCLC.

We have demonstrated that VEGFq has potent *in vitro* growth inhibitory activity in A549 cells (as well as other lung cancer cell lines), causing significant dose-dependent inhibition of cell proliferation starting 72 h after treatment. In contrast, control “mutated” oligonucleotides with a similar sequence, but which do not form quadruplex, have only a very slight growth inhibitory effect on NSCLC cells. Additionally, quadruplex-forming oligonucleotides targeted to the *HIF-1α* promoter have no growth inhibitory effect on A459 cells. Interestingly, the anti-proliferative effect of VEGFq on A549 cells could be partially reversed by the addition of recombinant VEGF protein confirming that VEGFq-mediated inhibition of cell proliferation is dependent on *VEGF* inhibition.

The VEGFq encoding oligonucleotide is remarkably resistant to nuclease degradation in serum and intracellularly and is abundantly taken into the nucleus and cytoplasm after 1 h relative to the MutVEGF oligonucleotide which does not form a G-quadruplex structure. Although VEGFq was also taken up by non-transformed Hs27 fibroblast cells, no change in growth occurred after 6 days suggesting that the growth inhibitory effects of VEGFq may be specific for transformed cells or cells overexpressing *VEGF* and that this oligonucleotide has no adverse effect on normal cells. These results are consistent with previous studies, which demonstrated that quadruplex-forming ODNs often show enhanced cellular uptake into tumor cells, form inherent nuclease resistant structures, and have minimal effects on non-transformed cells [20, 23, 24]. In general, G-quadruplex DNA has also been shown to accumulate and have greater retention in the nucleus compared to unstructured ODNs, which may be due to binding of specific targets inside the nucleus and/or G-quadruplex binding proteins.[45, 46]

Coincident with the anti-proliferative effects of the VEGFq oligonucleotide, treatment with VEGFq corresponded with a 50% decrease in cell invasion after 24h and attenuation of newly synthesized *VEGF* mRNA after 72 h and protein expression after 96 h and 6 days, which did not occur with MutVEGF treatment. This further demonstrates the specificity of VEGFq function; binding to its target sequence in the VEGF promoter, downregulating VEGF expression and subsequently, its activity. Previous studies have shown that VEGF stimulates endothelial cell invasiveness, which can be effectively inhibited with antisense VEGF oligonucleotides.[47]

Although VEGFq treatment inhibited A549 cell growth, no change in the cell cycle occurred after 96 h. This result is quite different from that obtained when leukemic cells were treated with the c-MYC promoter quadruplex-forming sequence, Pu27. Pu27 treatment caused G0/G1 cell cycle arrest in as little as 24h followed by an oncosis cell death.[20] Similarly, it has been shown that the “random” quadruplex-forming oligonucleotide, AS1411, induces cell cycle arrest in S Phase.[25, 48] In contrast, VEGFq treatment of A549 cells induced an autophagic cell death response, characterized by significant cytoplasmic accumulation of autophagic vacuoles and increased expression of LC3B. Although not definitive, these results are consistent with the different roles played by these two proteins in the transformed phenotypes of cancer cells, specifically the role of *VEGF* in cancer progression and the role of c-MYC in cell cycle regulation.[20]

The biological activity of VEGF is dependent upon its binding to VEGF receptor 1/2/3, with most of the downstream angiogenic activities transduced through VEGFR-2 by subsequent signaling through ERK 1/2 and AKT. However, VEGF can also bind to the more novel non-tyrosine kinase receptors, the Neuropilins (NP1 and NP2) to mediate its biological effects through VEGFR2-mediated cell signaling.[49, 50] The VEGF-activated protein kinases are frequently over-activated in a variety of cancers such as lung and play major roles in cancer cell proliferation, invasion/migration, and cell survival respectively.[51–55] Activated ERK can translocate to the nucleus and phosphorylate various transcription factors leading to altered gene transcription and cellular proliferation, while sustained AKT activation upregulates matrix metalloproteinases, which facilitate invasiveness.[12, 56] In NSCLC, ERK 1/2 and AKT activation is commonly associated with advanced and aggressive lung tumors and promotes chemotherapeutic resistance [57, 58]. It has been shown that down-regulation of *VEGF* decreases ERK1/2 and AKT activation in NSCLC, leading to pronounced inhibition of tumor growth, angiogenesis, invasion/migration, and promotion of cell death (apoptotic or autophagic)[59]. In our study, following treatment with VEGFq for 72 h, p-ERK 1/2 and p-AKT markedly decreased, despite unchanging levels of total ERK 1/2 and AKT protein expression suggesting that VEGFq inhibits cell growth and invasion through attenuation of the MAPK axis. In A549 cells, inhibition of AKT and the MAPK axis suppressed metastasis and induced autophagic cell death[60–62]. In hepatocellular carcinoma, inhibition of ERK phosphorylation with an anti-angiogenic retinoid effectively inhibited endothelial cell growth and migration[63]. In chronic myeloid leukemia, VEGF facilitated tumor proliferation and tissue angiogenesis of leukemic and endothelial cells, which was inhibited with antisense-*VEGF*[64]. The treatment of NSCLC cells with VEGFq resulted in significantly decreased VEGF expression, altering the downstream pathways which play important roles in tumor cell behavior, including proliferation/survival and invasion, most likely via blocking the autocrine-loop, but have minimal effects on non-transformed cells.

Currently, the relatively poor cure rate in lung cancer patients has been associated with resistance to chemotherapy and radiation due to innate characteristics of lung cancer cells. Novel treatment strategies such as VEGFq which induce cell death and reduce the availability of VEGF in the tumor milieu may be an alternative mechanism to more effectively inhibit growth of lung tumors [65–67].

## Conclusions

Our studies demonstrate that treatment of NSCLC cells with oligonucleotides encoding the VEGFq sequence inhibits cell growth and attenuates cell invasion through a mechanism involving inhibition of *VEGF* expression, downregulation of activated ERK 1/2 and AKT, and eventually induction of cell death. The demonstration of sequence-specific binding of the VEGFq oligonucleotide to its double stranded target sequence strongly supports the hypothesis that VEGFq binds to the genomic *VEGF* promoter by strand invasion in a sequence-specific (Watson-Crick) manner. This would, of necessity, stabilize the quadruplex structure formed by the G-rich strand of the genomic *VEGF* promoter, resulting in transcriptional inhibition of *VEGF* expression. It is evident that the cellular effects of VEGFq are quite different from those of Pu27, further supporting the gene-specificity of these oligonucleotides. This study suggests that the VEGFq oligonucleotide may represent a novel therapy for the treatment of NSCLC alone or in combination with standard lung cancer chemotherapies. This approach of gene-specific inhibition of transcription can be applied to the wide array of genes whose promoters contain quadruplex-forming sequences.

## Supporting information

S1 File Original data for Fig 8C. Fig A: Picture of the blot used to generate Fig 8B for VEGF expression. Fig B: Picture of the Blot used to generate Fig 8C for ERK and activated ERK expression. Fig C: Picture of the Blot used to generate Fig 8C for AKT and activated AKT expression.
(DOCX)
