# Supplementary material for: Quadruplex-forming oligonucleotide targeted to the VEGF promoter inhibits growth of non-small cell lung cancer cells
Source: PLoS One. 2019 Jan 25;14(1):e0211046. doi: 10.1371/journal.pone.0211046 (PMC6347295; doi:10.1371/journal.pone.0211046)
Supplement: S1 File — Fig A: Picture of the blot used to generate Fig 8B for VEGF expression. Fig B: Picture of the Blot used to generate Fig 8C for ERK and activated ERK expression. Fig C: Picture of the Blot used to generate Fig 8C for AKT and activated AKT expression. (DOCX) [file pone.0211046.s001.docx]

**Supplementary information**

Pictures of the original Western Blot gels used to generate the figure 8


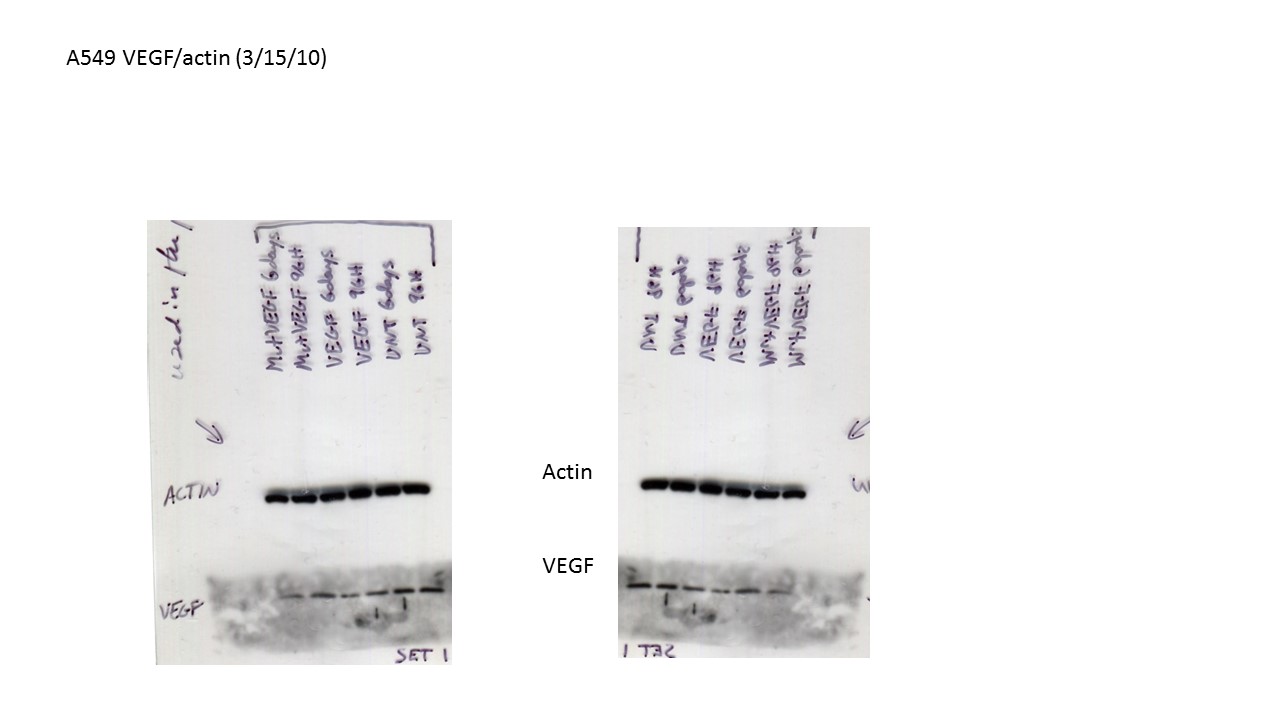


**Fig A: Picture of the blot used to generate Fig. 8B for VEGF expression:** A549 exposed to VEGFq, MutVEGFq or untreated for 96-144h. The same blot was probed with antibodies against VEGF and Actin.

On this blot, the samples were loaded from MutVEGF to untreated. For clarity and consistency of Fig. 8, the picture was rotated to have the untreated samples in line 1and 2, VEGFq treated in lines 3 and 4 and MutVEGF treated in lines 5 and 6.


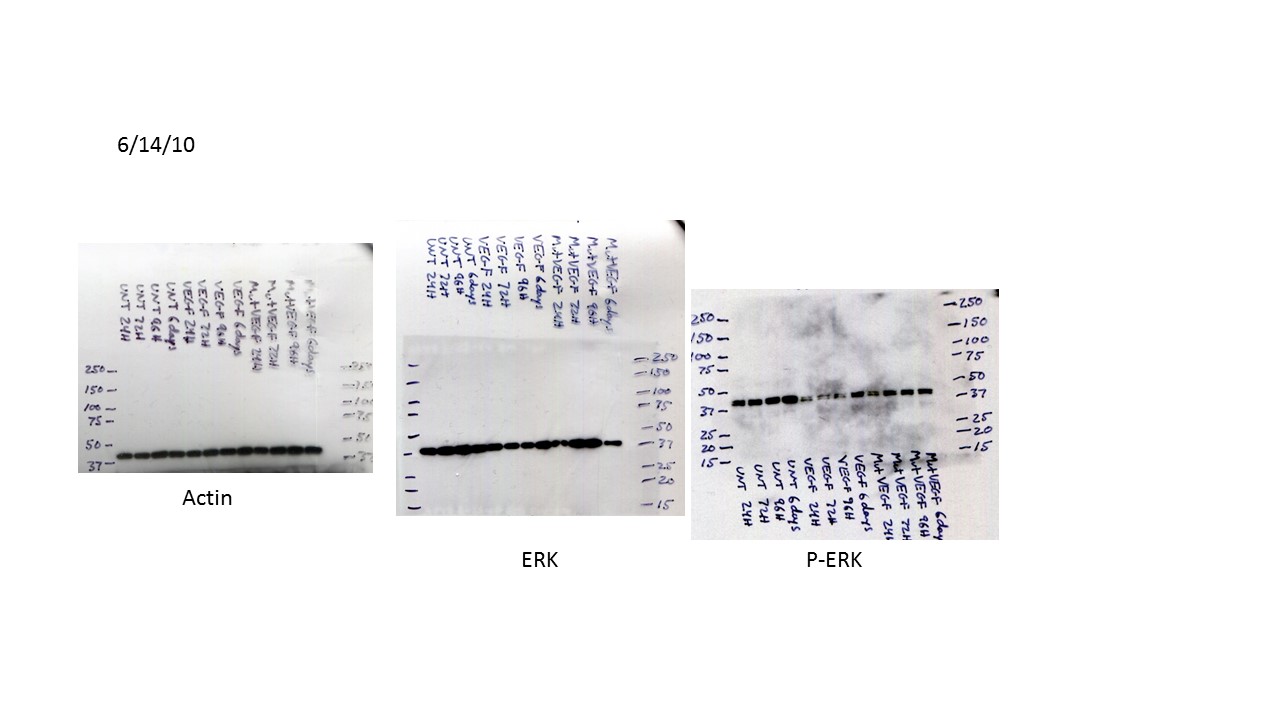


**Fig B: Picture of the Blot used to generate Fig. 8C for ERK and activated ERK expression.** A549 exposed to VEGFq or MutVEGFq or untreated for 24-144h. The same blot was probed with antibodies against ERK, phospho- ERK and Actin.


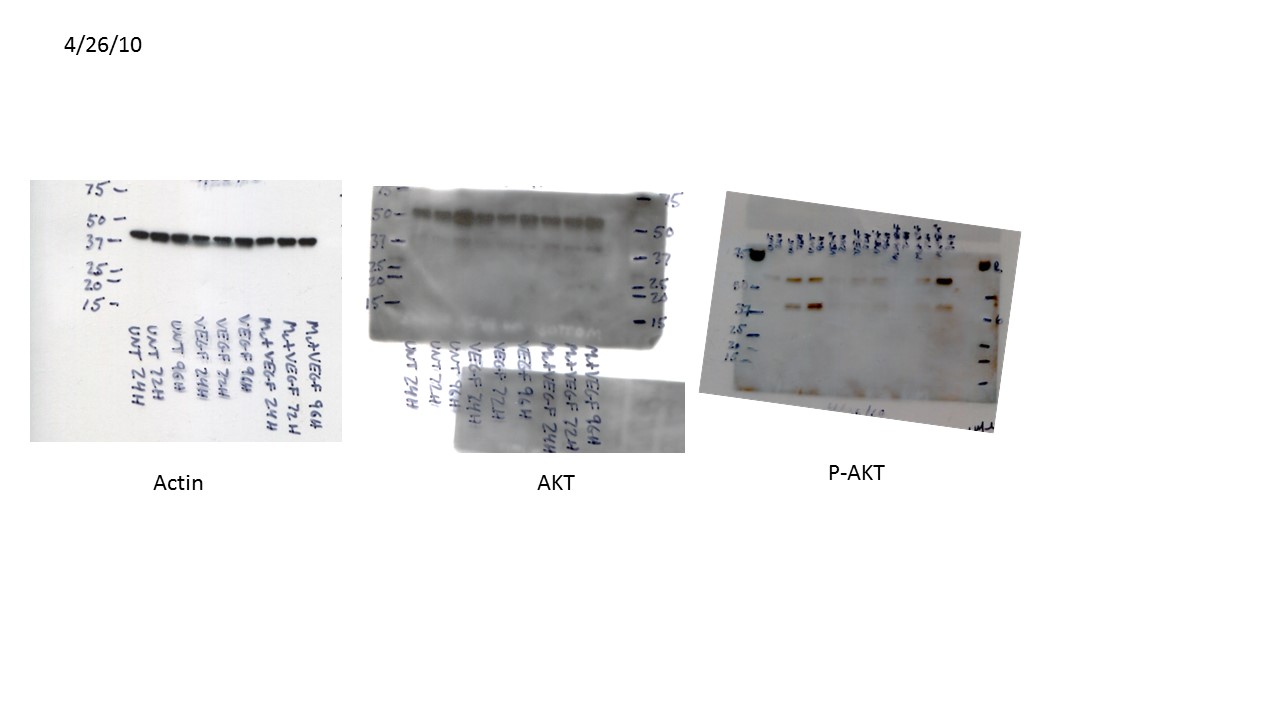


**Fig C: Picture of the Blot used to generate Fig. 8C for AKT and activated AKT expression.** A549 exposed to VEGFq or MutVEGFq or untreated for 24-96h. The same blot was probed with antibodies against AKT, phospho- AKT and Actin.
